# The elimination of trachoma as a public health problem in Mexico: From national health priority to national success story

**DOI:** 10.1371/journal.pntd.0010660

**Published:** 2022-08-29

**Authors:** Víctor Quesada-Cubo, Dey Carol Damián-González, Francisco Gibert Prado-Velasco, Nadia Angélica Fernández-Santos, Gustavo Sánchez-Tejeda, Fabián Correa-Morales, Hermilo Domínguez-Zárate, Abel García-Orozco, Martha Idalí Saboyá-Díaz, María Jesús Sánchez-Martín

**Affiliations:** 1 Independent Researcher, Madrid, Spain; 2 Trachoma Jurisdictional Coordination in San Cristóbal de las Casas, Chiapas, Mexico; 3 State Coordination of Trachoma in Chiapas, Chiapas, Mexico; 4 Instituto Politécnico Nacional, Centro de Biotecnología Genómica, Reynosa, Mexico; 5 Department of Entomology, Texas A&M University, College Station, Texas, United States of America; 6 Vector-Borne Diseases Program, National Center for Disease Control and Preventive Programs (CENAPRECE), Mexico City, Mexico; 7 Directorate of Public Health, Chiapas Ministry of Health, Chiapas, Mexico; 8 State Department of Vectors, Chiapas, Mexico; 9 Department of Communicable Diseases and Environmental Determinants of Health, Pan American Health Organization, Washington, D.C., United States of America; 10 Department of Communicable Diseases and Health Determinants, Pan American Health Organization, Mexico City, Mexico; Carter Center, UNITED STATES

## Abstract

**Introduction:**

Mexico was the first country in the Americas and the third in the world to eliminate trachoma as a public health problem, as validated by the WHO in 2017.

**Objective:**

To describe the critical elements that favored the elimination of trachoma as a public health problem in Mexico and the public health impact of this success.

**Methodology:**

A revision and compilation of data and information contained in the dossier presented by the country to PAHO/WHO to obtain the validation of trachoma elimination as a public health problem was conducted by a group of delegates from the national and local trachoma prevention and control program. Data from the national and local surveillance systems and reports of actions conducted after achieving the elimination goal were also included. Critical elements that favored the achievement of the elimination goal from 1896 to 2019 were extracted.

**Results:**

Mexico reached the elimination of trachoma in 2016 obtaining the validation in 2017. 264 communities were no longer endemic and 151,744 people were no longer at risk of visual impairment or possible blindness due to trachoma. The key to the success of this elimination process was primarily the local leadership of health authorities with sustained funding for brigades, increased access to potable water and sanitation, and key alliances with indigenous authorities, health authorities, and government institutions that contributed to the achievement of the goal. The SAFE strategy started implementation in Mexico in 2004 as a comprehensive package of interventions. SAFE stands for surgery, antibiotics, facial cleanliness, and improvement of the environmental conditions. These actions impacted drastically on the number of new cases trachmatous trichiasis (TT) and trachomatous inflammation-follicular (TF), which decreased from 1,794 in 2004 to zero in 2016.

**Conclusions:**

The elimination of trachoma as a public health problem in Mexico is a true success story that may serve as a model example for the elimination of other neglected infectious diseases in the Americas.

## Author summary

Trachoma is an eye disease caused by an infection with *Chlamydia trachomatis*. It is the principal infectious cause of irreversible blindness worldwide and is one of the 20 neglected tropical diseases which is present in 1,338 districts worldwide with an estimated 154.5 million people living in endemic areas. Globally, the disease was earmarked for elimination as a public health problem by the year 2020 and later extended to the year 2030. The WHO elimination strategy SAFE includes Surgery, Antibiotics, Facial cleanliness, and Environmental improvement. This paper describes the SAFE activities implemented and actions taken for Mexico to achieve the elimination of trachoma as a public health problem. From the compilation of national and local data and information, it was possible to identify that local and federal political commitment and leadership, effective coordination across health institutions and critical sectors such as water and sanitation agencies, and consistent communication and health promotion activities that supported the implementation of SAFE strategies were determinant in driving trachoma elimination. The model of coordinated work and collaboration serves as a model example for the elimination of trachoma and other Neglected Infectious Diseases in Latin America.

## Introduction

Trachoma is the leading infectious cause of blindness in the world and is caused by the bacteria *Chlamydia trachomatis*. The main risk factors for trachoma are largely preventable conditions including poor community hygiene, inadequate access to water, inadequate means for disposal of human feces, and overcrowding [1,2]. It is secondary to infection from *C*. *trachomatis*, which is transmitted between individuals in close proximity, spread from eye to eye by fingers, or by infected ocular and nasal secretions on fingers; it may also be transmitted by flies and fomites [1,2, 3,4,5,6]. Infection is associated with inflammatory changes of the conjunctivae known as Trachomatous Inflammation-Follicular (TF) and Trachomatous Inflammation–Intense (TI). Repeated episodes of TF and TI can result in scarring of the eyelid, which in some individuals leads to trichiasis (one or more eyelashes are pulled inwards to touch the eye) with entropion (in which the eyelid margin is rolled inwards). If trichiasis is left untreated, it may lead to corneal opacification, low vision, and blindness [7,8].

Epidemiologically today, trachoma continues to affect poor, vulnerable, isolated populations and it is endemic in many of the poorest and most rural areas of Africa, Central and South America, Asia, Australia and the Middle East. Trachoma especially affects young children and women in 44 endemic countries worldwide [9] where it is estimated that 136 million people are at risk of blindness. Over 20 million have active trachoma, 1.9 million are blind (it causes about 1.4% of all blindness worldwide), and 8.9 million require surgery.

**Fig 1:**
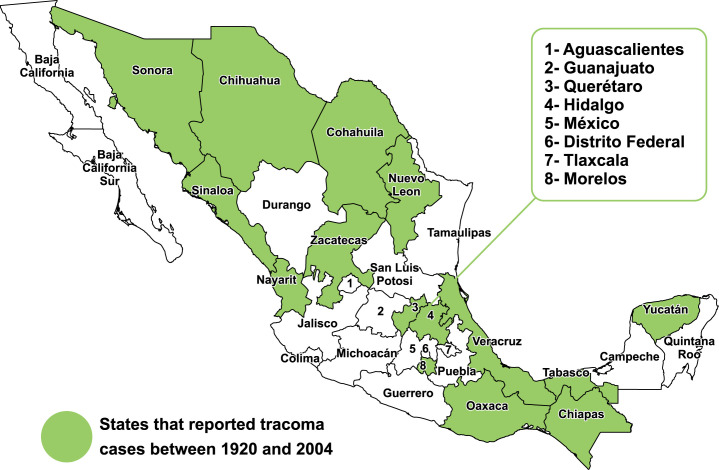
Report of cases of trachoma in Mexico. 1920–2004. Resource: Own elaboration based on data provided by Dr. Sheila West and extracted from dossier [13].

In the Americas, trachoma has been reported as endemic in recent years in Brazil, Colombia, Guatemala, and Peru [10,11]. Trachoma had a wide distribution in Mexico as obtained from historical records [12], and the state of Chiapas always concentrated the highest number of cases such that in 1876 there was evidence of at least 42,000 cases of trachoma. Various studies carried out in Chiapas showed a 50% prevalence of trachoma cases (classified as active and chronic cases), with Oxchuc and Chanal (80%), Tenejapa (60%) and Huixtan (20%) being the most highly endemic municipalities **(Fig 1)**.

In May 2022, 15 countries have reported achieving elimination goals: but only thirteen of those countries: Cambodia, China, Gambia, Islamic Republic of Iran, Lao People’s Democratic Republic, Ghana, Mexico, Morocco, Myanmar, Nepal, Oman, Saudi Arabia and Togo had been validated by WHO as having eliminated trachoma as a public health problem [9].

The purpose of this report is to describe the historical process and the key elements that favored the achievement of the elimination of trachoma as a public health problem in Mexico.

## Methodology

The main source of data and information presented was from trachoma elimination dossier, compiled by the Ministry of Health of Mexico, called dossier [13], and submitted to PAHO/WHO for validation of trachoma elimination.

The dossier contained a compilation of historical information related to the trachoma elimination process and it was completed by the Ministry of Health in collaboration with several public and private organizations and partners in the country. The dossier has information from the following sources:

1. Historical documents: a bibliographic review of reports including official documents of the Trachoma Program (national and subnational), conference proceedings on the subject, as well as gray literature (interviews, documentaries, and others) describing the history of the disease in Mexico and Chiapas and the epidemiology of the disease throughout the years (1896–2019).
2. Reports and internal documents of the National Trachoma Prevention and Control Program: description of strategies and activities carried out by the Trachoma Brigades (record of activities, comprehensive health programs, and indicators) with special emphasis on the Trachoma Program in Chiapas.
3. Epidemiological information on trachoma reported through the health information system: this included reports of detected cases, epidemiological case investigation forms, case series, and others from the National Secretary of Health.
4. Official reports containing the results of the evaluation of the interventions, validation, and post-validation data of the PAHO/WHO.

The information in the dossier and the other sources of information was reviewed by a multidisciplinary team of professionals made up of national and international experts, who conducted a critical analysis of the most relevant information. They also reviewed the accuracy of the information according to the sources referenced in the dossier. The information was organized chronologically and a description of the process and key elements that made the country eliminate trachoma were described. The information on the elimination process in trachoma endemic municipalities of Chiapas was organized by each of the four pillars of the SAFE strategy.

Permission to conduct the original data compilation was obtained from the Secretary of Health in Mexico.

## Results

### Trachoma in Mexico

Trachoma as an infectious cause of blindness was first reported in Mexico in 1896 when the Ophthalmology Hospital had documented over 40,000 trachoma cases among vulnerable indigenous individuals from the Toluca and Texcoco valleys in central Mexico [14]. This was mainly due to the large amount of immigration attracted to large construction project that were carried out during the Profirio Diaz regime, among which we must highlight the railroad lines, for which a lot of labor was required.

Although the conditions in the indigenous towns of the Toluca Valley and Texcoco were generalized in the country, trachoma was evident in this area due to the number of cases that were attended at the Ophthalmology Hospital among other reasons because there were trained personnel to recognize this new situation.

**Fig 2:**
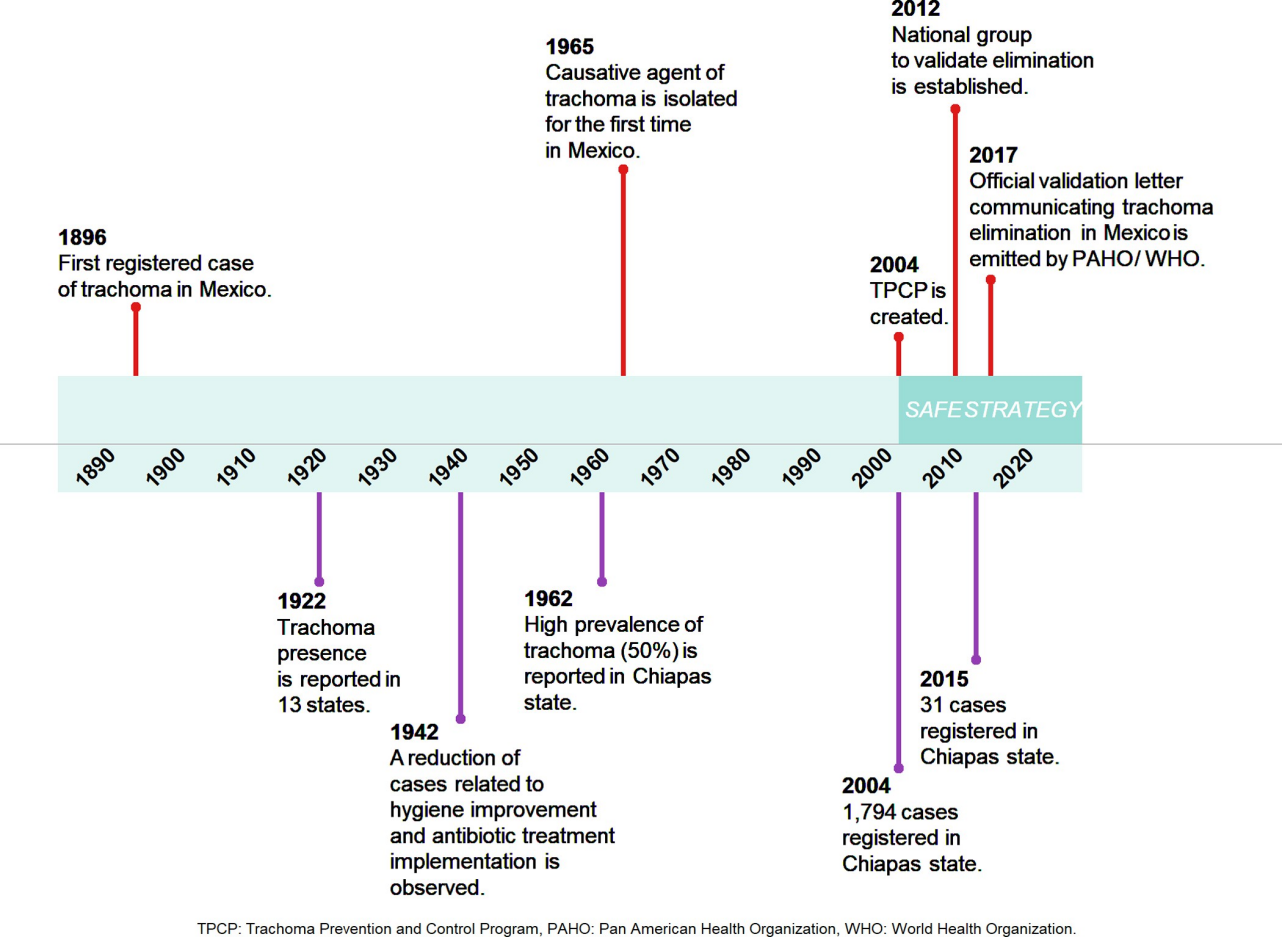
Milestones in the trachoma elimination in Mexico, 1890 to 2020. Source: Own elaboration based on extraction of data from dossier [13].

By 1906 social and environmental determinants of the disease were established reporting overcrowding, insufficient hygiene and sanitation as well as the influx of migrants cause that favored transmission of the disease [12,15]. The main milestones can be seen in the following timeline **(Fig 2).**

**Fig 3:**
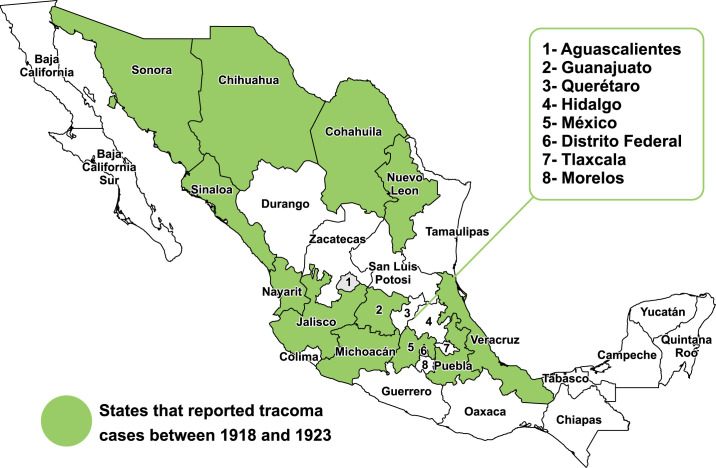
States with reports of trachoma in Mexico, 1918–1923. Source: Own elaboration based on analysis data provided Vélez D. and collaborators [12].

Ophthalmologists and health authorities throughout the country reported the presence of trachoma cases in 13 states: Sonora, Michoacán, Puebla, Veracruz, Chihuahua, Coahuila, Nuevo León, Sinaloa, Nayarit, Jalisco, Guanajuato, Mexico, and Chiapas between 1918 and 1923 [12] **(Fig 3).** This activity was part of the national surveillance system that included the compulsory reporting of trachoma cases.

By 1942 a reduction in cases was observed, attributable to the implementation of control measures and treatment with antiseptic solutions, surgical curettage, the use of antibacterial sulfonamides, and the construction of the hospital network in México [16].

Between 1944 and 1961 there were no reports published of trachoma in Mexico due to structural changes occurring in the national health system [17]. In 1962, ophthalmologist Dr. Javier Torroella, while visiting Chiapas, [18] reported a high prevalence (50%) of trachoma among *Tzeltal* indigenous population in the municipalities of Oxchuc, Chanal, Cancuc, Huixtan, Tenejape, Tenengo (Ocosingo municipality) and Guaquitepec (Chilón municipality), with a total population of 60,000 people; the most affected municipality was Oxchuc where it was estimated that 10% of its population was blind. As an immediate response to Torroella’s direct report to the Secretary of Health, health centers were built in Oxchuc and Chanal, and the manufacture and distribution of tetracycline ointment for treatment were implemented. In 1965 the causative agent of trachoma is isolated for the first time in Mexico [18]. Between 1998 and 2001 the prevalence of trachoma in the general population was 20% in 21 communities in the endemic area of Chiapas.

For about 30 years (1970’s– 1990’s), limited trachoma studies, prevention, and control activities were conducted in Chiapas [17]. Since 1995, all suspected cases of trachoma were evaluated by WHO-certified Trachoma Brigade examiners and the epidemiological investigation was completed to confirm or discard cases. Confirmed cases were reported through the national surveillance system **(Table 1)**.

**Table 1:** Trachoma suspected cases identified by State in Mexico, 1923 to 2015. Source: General Directorate of Epidemiology and Directorate of Vector-Borne Diseases of CENAPRECE and literature review of trachoma in the Americas by PAHO [13].

State	Reports and Publications (Year)	1995	1996	1997	1998	1999	2000	2001	2002	2003	2004	2005	2006	2007	2008	2009	2010	2011	2012	2013	2014	2015	Total
Aguascalientes		3	0	0	1	0	0	0	0	0	0	0	0	0	0	0	0	0	0	0	0	0	4
Baja California		1	0	1	0	0	0	0	0	0	0	0	0	0	0	0	0	0	0	0	0	0	2
Baja California Sur		0	0	0	0	0	0	0	0	0	0	0	0	0	0	0	0	0	0	0	0	0	0
Campeche		0	0	0	0	0	0	0	0	0	0	0	0	0	0	0	0	0	0	0	0	0	0
Coahuila	1923 and 1983	0	1	0	1	0	0	0	0	0	0	0	0	0	0	0	0	0	0	0	0	0	2
Colima		4	0	0	0	0	0	0	0	0	0	0	0	0	0	0	0	0	0	0	0	0	4
Chiapas	Asian migrants (1923)	23	38	0	4	35	0	0	0	0	1,794	760	631	228	113	31	82	96	36	85	59	33	4,048
Chihuahua	1923	2	0	0	0	1	0	0	0	0	0	0	0	0	0	0	1	0	0	0	0	0	4
Distrito Federal		6	0	1	1	0	0	0	0	0	0	0	0	0	0	0	0	0	0	0	0	0	8
Durango		0	0	11	4	0	0	0	0	0	0	0	0	0	0	0	0	0	0	0	0	0	15
Guanajuato	1923	0	0	0	0	0	0	0	0	0	0	0	0	0	0	0	0	0	0	0	0	0	0
Guerrero		16	12	1	0	0	0	0	0	0	0	0	0	0	0	0	0	0	0	0	0	0	29
Hidalgo	2003	0	0	0	0	0	0	0	0	0	0	0	0	0	0	0	0	0	0	0	0	0	0
Jalisco	1923	0	0	1	1	0	0	0	0	0	0	0	0	0	1	0	0	0	0	0	0	0	3
México	1906 and 1923	0	0	3	1	0	0	0	0	0	0	0	0	0	0	0	0	0	0	0	0	0	4
Michoacán	1923	0	1	0	1	0	0	0	0	0	0	0	0	0	0	0	0	0	0	0	0	0	2
Morelos		2	0	0	0	0	0	0	0	0	0	0	0	0	0	0	0	0	0	0	0	0	2
Nayarit	1923 y 2003	0	0	0	0	0	0	0	0	0	0	0	0	0	0	0	0	0	0	0	0	0	0
Nuevo León	1923	0	0	0	0	0	0	0	0	0	0	0	0	0	0	0	0	0	0	0	0	0	0
Oaxaca	2007	1	0	1	0	0	0	0	1	0	0	0	0	0	0	0	2	0	0	0	0	0	5
Puebla	1923	1	0	0	0	0	0	0	0	0	0	0	0	0	0	0	0	0	0	0	0	0	1
Querétaro	2003	1	0	0	0	0	0	0	0	0	0	0	0	0	0	0	0	0	0	0	0	0	1
Quintana Roo		0	0	0	0	0	0	0	0	0	0	0	0	0	0	0	0	0	0	0	0	0	0
San Luis Potosí		0	0	0	0	0	0	0	0	0	0	0	0	0	0	0	0	0	0	0	0	0	0
Sinaloa	1923 and 2003	0	0	0	0	2	0	0	0	0	0	0	0	0	0	0	1	0	0	0	0	0	3
Sonora	1923 and de 1931–1949	1	0	0	0	0	0	0	0	0	0	0	0	0	0	0	0	0	0	0	0	0	1
Tabasco		5	0	0	0	0	0	0	0	0	0	0	0	0	0	0	0	0	0	0	0	0	5
Tamaulipas		0	0	0	0	0	0	0	0	0	0	0	0	0	0	0	0	0	0	0	0	0	0
Tlaxcala		1	0	0	0	0	0	0	0	0	0	0	0	0	0	0	0	0	0	0	0	0	1
Veracruz	1923	18	5	0	0	0	0	0	0	0	1	0	0	0	0	0	0	0	0	0	0	0	24
Yucatán	1920 and 1931 (Asian migrants)	12	0	0	1	0	0	0	0	0	0	0	0	0	0	0	0	0	0	0	0	0	13
Zacatecas	2007	1	0	2	1	0	0	0	0	0	0	0	0	0	0	0	0	0	0	0	0	0	4
**Nacional**		**98**	**57**	**21**	**16**	**38**	**0**	**0**	**1**	**0**	**1,795**	**760**	**631**	**228**	**114**	**31**	**86**	**96**	**36**	**85**	**59**	**33**	**4,185**

**Fig 4:**
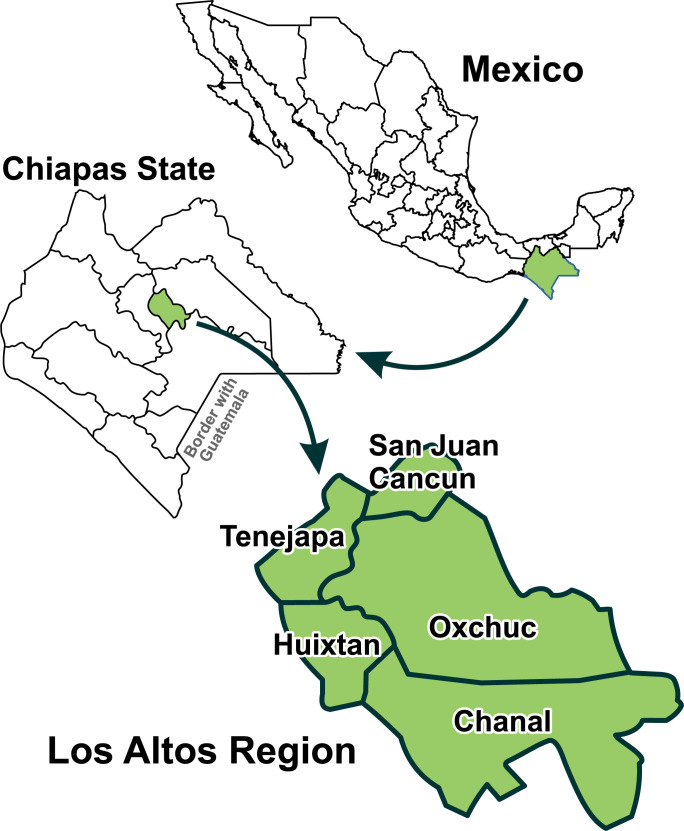
Trachoma endemic municipalities in the state of Chiapas. 2015. Source: Own elaboration based on data from Ochoa H. and collaborators [13].

By 2016, the known trachoma area in Mexico was established in Altos de Chiapas, encompassing the municipalities of Chanal, Huixtan, Oxchuc, Tenejapa, and San Juan Cancuc [19,20,21], concluding that this was the only area in Chiapas and Mexico with trachoma [17] **(Fig 4)**.

### Trachoma prevention and control program (TPCP) in Chiapas

It was not until the beginning of the 21^st^ century that more aggressive actions were implemented to control trachoma in the five endemic municipalities (246 communities) with known cases in Chiapas. The Chiapas Secretary of Health also recognized the importance of the control and elimination of trachoma as a public health priority for the state and in 2004 the Chiapas Institute of Health created the Trachoma Prevention and Control Program (TPCP). This program became a political priority, attracting local, national, and international resources to support the program [13].

The main strategy of the TPCP was the creation and implementation of “The Trachoma Brigades” in the 5 endemic municipalities, with approximately 60 (at its highest point) doctors, nurses and health technicians that were trained to carry out clinical detection of trachoma cases, through house-to-house visits twice a year. Upon detecting a case in the active phase, they provided treatment with azithromycin to cases and contacts and promoted family facial hygiene. Detected cases of TT were offered corrective surgery and a brigade member carried out the post-operative follow-up of each operated case. A network of 415 teachers was also trained and involved together with the community workers exclusively dedicated to promoting trachoma prevention and control with funding provided by the local, state, and federal governments.

A stakeholders’ network for the control of trachoma was established and consolidated between 2000 and 2006. They met every trimester and included collaboration across public sectors such as agriculture, education, academic institutions, local and national water commission as well as several initiatives from the private sector were included all with intense interventions on community health promotion activities with the participation of local indigenous leaders and traditional healers and carrying out numerous health promotion events on regular bases [13].

An important water project was also implemented in 2006 by the Mexican Institute of Water Technology and the National Water Commission to improve access to water supply in identified endemic communities [14]. By 2009, only 50.1% of the population in the endemic communities and 66.2% of schools had piped water in their homes and premises, respectively. The Hydraulic Plan had assigned a budget of close to 13 million dollars by 2010, aimed at both rural and urban areas, to install drinking water piping networks, sewerage networks, latrines, and garbage collection **(Table 2).** Water filters using silver ions and water tanks of 750 liters were also distributed to families in the endemic communities [14].

**Table 2:** Investment in basic water and sanitation in trachoma endemic municipalities, Chiapas State, 2006–2012. Source: Sustainable Plan for Hydraulic Works, Sanitation and Responsible Treatment of Solid Waste. Government of the State of Chiapas. 2006–2010.

	Endemic Municipalities							
Intervention	Chanal	Huixtán	Oxchuc	San Juan Cancuc	Tenejapa	Total	Entry	Investment (USD$)
Water treatment plant	10	14	8	13	26	**71**	Drinking water	724,611.71 USD
Containers	2	2	5		2	**11**	Drinking water	274,790.92 USD
Water System	4	8		4	4	**20**	Drinking water	1,998,159.96 USD
Ecological latrine	206		388	612	744	**1**,**950**	Sanitation	1,924,114.22 USD
RWTP*		2	1	3	1	**7**	Sanitation	3,536,068.96 USD
Construction Works		1	2			**3**	Drinking water	486,427.36 USD
Sewage system		8	6		8	**22**	Sanitary sewer	2,314,487.57 USD
Water tank			57			**57**	Drinking water	29,868.58 USD
Storage Tank				4		**4**	Drinking water	418,160.10 USD
Planning**			1		1	**2**	Sanitation	26,881.72 USD
Water system and water treatment plant					3	**3**	Drinking water	943,847.07 USD
Sanitation system					2	**2**	Sanitation	102,846.06 USD
Ecological latrines					2	**2**	Sanitation	219,343.07 USD
Total construction sites	222	35	468	636	793	**2**,**154**		
Total investment grouped by municipality	642,609 USD	2,566,090 USD	2,329,616 USD	2,340,546 USD	5,120,744 USD	**12,999,607 USD**		12,999,607 USD
Total number of inhabitants benefited	10,681	15,045	32,324	41,796	49,488	149,334		

*RWTP: Residual water treatment plant

**Planning: projects and works planning

By 2004, Chiapas implemented the SAFE strategy, based on Mexico’s commitment as the Alliance for Global Elimination of Trachoma by 2020 (GET2020) [22] and the resolution WHA51.11 calling for increased implementation of the SAFE strategy to support the elimination of trachoma worldwide [23].

### Implementation of SAFE in Chiapas

The Trachoma Brigades visited 246 endemic municipalities conducting house-to-house and school visits twice a year between 2004 and 2016, for active searching of trachoma cases, treatment, follow-up, and data recording on housing and individual risk factors.

#### 1) Surgery (S)

Field personnel in the Trachoma Brigades were trained to identify trachomatous trichiasis (TT) cases and if the patient was a surgery candidate they were informed of the treatment alternative. Those that accepted were included in the program plan for Surgery using the Bilamellar Rotation technique performed by WHO/PAHO certified surgeons. From 2012 to 2017, TT surgery was only performed in the Capital City of Chiapas, Tuxtla Gutierrez (Chiapas) by oculoplastic surgeons supporting the Trachoma Program. These surgeons continue supporting the Trachoma Program in the post-validation phase. All patients received 1gr of oral azythromicyn preoperation, and postoperation antibiotic was prescribed based on surgeons’ discretion. To improve compliance and acceptance of surgery, several actions were taken in 2012 to support the patients economically: transportation to surgery sites with family members as needed, food and lodging, designation of a brigade member to manage patient care throughout the whole process, including post-surgical follow-up visits, supplies to promote facial hygiene, and post-surgery antibiotics provided free of charge by the National Health System.

People refusing surgery after being offered counseling repetitively (at least 5 times) had to sign a document to that effect confirming their decision, and a brigade member had to record this decision on up to five separate visits.

Between 2004 and 2015, a total of 487 TT surgeries were performed [13]. After surgery, each patient was provided with supplies for wound cleaning and followed-up on day 3, day 7, and 1 year **(Table 3)**. A total of 146 patients that were identified at early stages of TT and had had epilation using electrolysis were monitored and later operated on due to the evolution of the entropion.

**Table 3:** TT operations carried out in Chiapas, Mexico, 2004–2015. Source: State Program for the Prevention and Control of Trachoma.

Year	Oxchuc	San Juan Cancuc	Tenejapa	Huixtán	Chanal	Total
2004	1	4	0	2	0	7
2005	12	4	3	3	1	23
2006	40	17	4	8	5	74
2007	8	0	4	0	0	12
2008	1	0	4	0	1	6
2009	39	0	9	11	0	59
2010	30	0	0	4	0	34
2011	7	0	2	0	0	9
2012	8	0	1	1	2	12
2013	99	14	17	11	8	149
2014	9	25	28	29	2	93
2015	0	0	0	9	0	9
**Total**	301	50	56	67	13	487

#### 2) Antibiotics (A)

The TPCP personnel carried out house-to-house visits every 6 months to all the communities in the five trachoma endemic municipalities examining all residents looking for trachoma cases. If trachoma active cases were detected, they and their contacts were treated with a single dose of azithromycin at the WHO recommended dose of 20 mg/kg. The Trachoma Program established an efficient supply and distribution network, so the antibiotic was available to all field Trachoma Brigades at all times and only the brigades handled the medicine. Azithromycin was purchased by the state of Chiapas, with PAHO support for importation. Treatment was given to all children 1 to 11 years old with TF able to swallow medication, and as prophylaxis to all people living in the same household, close contacts, and relatives including close neighbors. Exceptions were made for pregnant or breastfeeding women, children under 1 year of age, people with allergies, and those that were intoxicated at the time of the visit. The brigade’s health promotion activities included education on the importance of taking the antibiotic, its benefits and side effects, as well as the date of the next visit, ophthalmological examination, and evaluation. The communities were visited twice a year and the follow-up visits of the Trachoma Brigades continue to date.

A total of 6,333 doses of azithromycin were distributed throughout the five endemic municipalities from 2004 to 2014. From 2009 to 2014 an average of 5.9 treatments were administered to close contacts of each identified case with variations between years and municipalities **(Table 4).**

**Table 4:** Azithromycin doses distributed to trachoma active cases and contacts registered by State Trachoma Prevention and Control Program (PEPCT) by endemic municipality, State of Chiapas, 2004–2014. Source: State Program for the Prevention and Control of Trachoma.

Municipality	2009			2010			2011			2012			2013			2014		
	Case	Tx	Hc	Case	Tx	Hc	Case	Tx	Hc	Case	Tx	Hc	Case	Tx	Hc	Case	Tx	Hc
**Chanal**	**0**	0	0.0	3	42	0.0	3	27	0.0	0	0	0.0	3	16	0.0	5	29	0.0
**Huixtán**	**0**	0	0.0	2	15	0.0	7	26	0.0	0	0	0.0	3	12	0.0	3	28	0.0
**Oxchuc**	**9**	67	6.4	8	119	13.9	17	97	4.7	5	8	0.6	6	59	8.8	2	13	5.5
**Tenejapa**	**2**	26	12.0	26	210	7.1	25	235	8.4	4	17	3.3	29	58	1.0	25	176	6.0
**San Juan Cancuc**	**5**	36	6.2	9	35	2.9	9	65	6.2	13	85	5.5	9	77	7.6	13	130	9.0
**Total**	**16**	**129**	**7.1**	**48**	**421**	**7.8**	**61**	**450**	**6.4**	**22**	**110**	**4.0**	**50**	**222**	**3.4**	**48**	**376**	**6.8**

Tx–Treatment dose

Hc- Houshold contacts

#### 3) Facial cleanliness (F)

The TPCP through the Trachoma Brigades has implemented facial cleanliness promotion campaigns in schools and households in the endemic area since 2004. Initially, the “Regional Week to Combat Trachoma” was created to promote facial hygiene in schools. These weeks were supported by bilingual staff, local health authorities, and social mobilization through events in the five trachoma endemic municipalities and communities. Cultural events, parades, puppet theater, plays, poster campaigns, face, and hand washing workshops, and posters to inform the population were included. Sometimes they were also accompanied by surgical campaigns for TT patients in the municipalities. Since 2005, they included other means of promotion such as television, radio, videos, posters, manuals, flipcharts, and workshops where community health workers and health promoters became highly involved. In 2010, the Trachoma Brigades distributed soap and towels in schools of the five municipalities to facilitate face washing, which was reinforced by promoting the strategy “I wash my face” among school teachers and parents of children between 1 and 9 years of age in 2014. After the elimination of onchocerciasis in Mexico in 2015, the health workers of this program were integrated into the Trachoma Program to strengthen health promotion in communities and schools.

Between 2010 and 2014, the strategy of facial cleanliness was evaluated in children under 11 years old in the endemic municipalities with encouraging results. In order to improve adherence, a tool adapted to children’s capacity for expression was designed, which is based on a graphic visual scale with three figures of face washing that represented how the face looks after washing, evaluating the cleanliness as "good", "regular" or "bad" and in which the children marked the one with which they felt most identified. In 2010, 43,026 children were evaluated and 97.4% of them had satisfactory levels of cleanliness. By 2014, this percentage had increased to 99.3% (44,059 children) **(Table 5)**.

**Table 5:** Assessment of facial hygiene in municipalities known to have trachoma in Chiapas. 2012–2014. Source: State Program for the Prevention and Control of Trachoma.

Municipality/Children	Clean face			With eye discharge			With nasal discharge			Dirty face		
	2012	2013	2014	2012	2013	2014	2012	2013	2014	2012	2013	2014
Chanal	0	3,630	3,025	0	1	2	0	28	17	0	31	24
Huixtan	0	4,413	5,012	0	30	0	0	8	0	0	13	2
Oxchuc	15,202	14,855	14,398	25	22	18	96	68	79	108	67	46
San Juan Cancun	10,555	8,112	10,734	42	1	7	68	21	67	195	22	25
Tenejapa	2,167	5,643	10,890	12	3	2	24	40	20	50	34	3
**Total**	27,964	36,653	44,059	79	57	29	185	165	183	353	167	100

#### 4) Environmental improvement (E)

Environmental improvement for the reduction of transmission of trachoma includes several interventions. The Chiapas State government considered that an important contribution to the population’s well-being was to increase access to safe water supply and sewage disposal for the population in the 28 municipalities with very low human development index, which included the five trachoma endemic municipalities where only 58% of the population had access to piped water compared to the 87.1% national average. The National Water Commission of Mexico (CONAGUA) in coordination with the Chiapas Health Secretary and the TPCP established in 2005 a project to increase access to piped water that reached 467,264 houses in 3,825 communities in rural and urban areas. The Trachoma Program, as well as other associations and institutions involved, were instrumental in persuading state and federal authorities of the need to provide these benefits to the population. In 2009 alone, the Hydraulic Plan was established, aimed at both rural and urban areas with the following programs Water, Sewerage and Sanitation in Urban Areas (APAZU) and the agreement for the construction and rehabilitation of drinking water and Rehabilitation of Drinking Water and Sanitation Systems in Rural Areas (PROSSAPYS). This project was an investment of USD 131 million. In the same year, Rotary Club International distributed filters for the purification of water in endemic municipalities [13]. Between 2006 and 2012, a total of USD 13 million was invested in basic sanitation and drinking water storage reaching 149,334 people in the trachoma endemic municipalities [24].

Environmental improvements also included the building of new houses carried out by Fideicomiso Fondo Nacional de Habitaciones Populares (FONHAPO) with a total investment of USD 4 million between 2013 and 2015 [13]. The TPCP reported in 2012 that only 0.4% of the 26,269 houses in the endemic areas did have pit latrines. Environmental sanitation interventions improved the living conditions of 149,334 people (98%) in the endemic area.

### Trachoma rapid assessment outside Chiapas

For over 30 years there was no evidence of trachoma outside Chiapas in Mexico. Based on social development and health conditions, a trachoma vulnerability index was created for 40,592 rural communities, assuming that those with the highest index were more vulnerable, which may mean that they are at greater risk of ocular trachoma.

The vulnerability index comprised the combination of five indicators [13]: 1) Frequency of inhabited private dwellings that do not have a toilet, latrine, or cesspool, 2) Frequency of inhabited private dwellings that do not have access to drinking water, faucet or hydrant, from another dwelling, from a pipe, from a well, river, stream, lake, or other; 3) Frequency of private inhabited dwellings with dirt floors; 4) Frequency of indigenous population, and 5) Frequency of people with difficulty seeing, even when wearing glasses.

Nine communities with the highest vulnerability indices were selected to conduct a trachoma rapid assessment [25]; three communities in each of the States of Guerrero, Nayarit, and Oaxaca. In April 2016, the assessment was carried and 50 children between 1 and 9 years old were examined for active trachoma signs (Trachomatous Inflammation-Follicular (TF) and/or Trachomatous Inflammation-Intense (TI)) and adults aged 15 years and above were interviewed and examined for Trachomatous Trichiasis (TT). No trachoma cases were identified. It was concluded that if there were no trachoma cases in the most vulnerable communities assessed it was unlikely to have trachoma in other populations in the country. Based on these findings, it was considered that no additional assessments or surveys were needed and that trachoma had been eliminated as a public health problem in the country.

### Evaluation of interventions

Between 2000 and 2015, the General Epidemiology Directorate reported in Mexico a total of 3,844 confirmed trachoma cases from the known endemic municipalities of Chiapas. There was a steady decrease in all clinical forms from 1,794 in 2004 to only 31 in 2015. Subsequently, there were isolated cases that did not pose a public health problem.

All endemic municipalities reported TF prevalence of less than 5% in children aged 1–9 years old in 2014, the highest prevalence during this period 2009–2014 was recorded in the municipality of Tenejapa, which was reported in 2013 and 2014 a prevalence close 0,0025%.

To verify the absence of trachoma in other municipalities in Chiapas state, the TPCP carried out a trachoma survey in municipalities with no recorded cases. For that purpose, a random sample of 36 communities of the 112 municipalities of Chiapas was selected for surveillance in 2015. A total of 1,219 households were visited and 4,212 persons of 1 year of age and above were examined, distributed in three age groups a) 1–9 years old (2,038 people: 48%); b) 10–39 years old (1,470 people: 35%) and c) 40–96 years old (704 people: 17%).

Nine cases of TF were identified for an overall prevalence of 0.4% in children aged 1–9 years, no eye swab samples were identified as positive for *C*. *trachomatis*, and no TT cases were identified. All samples were sent to the international *C*. *trachomatis* laboratory at Johns Hopkins University for molecular testing.

### Validation of the elimination of trachoma

In August 2012, the National Group to validate the elimination of trachoma as a public health problem was set up to gather the historical, epidemiological, and programmatic information as evidence of the achievement of the elimination goals, and prepare the dossier. Mexico met the international criteria for the elimination of trachoma as a public health problem in 2016, namely [26]: (i) prevalence of TT unknown to the health system below 0.2% in adults aged 15 years and above; (ii) prevalence of TF below 5% in children aged 1–9 years in each formerly endemic district and (iii) proven evidence that the health system can identify and manage cases of TT under a defined protocol, with evidence of appropriate financial resources. The dossier prepared by the National Center for Disease Control and Preventive Programs (CENAPRECE), provided all requested information on the epidemiological data that supported the elimination criteria. The Ministry of Health established the National Validation Committee made up of national and international organizations delegates, with the task of reviewing the dossier and the information provided for its submission to PAHO/WHO. A formal request to validate the achievement and the dossier was officially submitted to the PAHO/WHO in 2016 [13].

On 3 January 2017, the WHO sent the official validation letter to the country that trachoma had been eliminated from Mexico as a public health problem, becoming the first country in the Americas to reach this goal, and the third in the world after Oman and Morocco to receive official validation [27].

### Post-validation activities

The TPCP continues the epidemiological surveillance of active cases and the implementation of the SAFE strategy in the five former endemic municipalities in Chiapas, although house-to-house searching of trachoma cases in these municipalities was suspended in 2017 and 2018 since the Trachoma Brigades were diverted to support other health priorities in Chiapas State. In 2019, efforts were made to visit the former endemic communities and a total of 200 people were followed-up by the Trachoma Brigades out of which nine were identified with TF and 27 with TT all in the municipality of Oxchuc. Sixty-four people were treated with azithromycin and 88 communities were visited for health promotion purposes. Twelve TT cases were operated by certified ophthalmologists in the Health Center of Oxchuc and the Woman Hospital in San Cristobal de las Casas. Surgeries for TT cases are free of charge for the affected people. Public hospitals are in charge of providing the surgery service which is performed by a surgeon employed by the Ministry of Health and trained by PAHO/WHO.

## Discussion

The case of Mexico is an example of how intersectoral, national, regional, state, and local work, led by the Health Sector, is capable of creating and effectively implementing public policies that allowed the identification of a health problem and its resolution. This report presented a summary of the strategies and actions implemented in trachoma endemic municipalities in Mexico to achieve elimination and to become the first country in the Americas to eliminate this disease as a public health problem in 2016 with the validation granted in 2017 [26,28].

By achieving this milestone, Mexico improved the quality of life of populations living in the former trachoma endemic municipalities by avoiding visual impairment and eventually blindness due to this disease [29].

The elimination of a neglected tropical disease such as trachoma in a country is the result of the unique combination of efforts at several levels, involvement of multiple stakeholders in close collaboration with the affected communities, domestic mobilization of funding to implement and sustain interventions including intersectoral efforts to improve living conditions of populations. This is a long process in which overcoming challenges is fundamental to sustaining the efforts made over centuries as described in this report for Mexico.

The following were the key elements that favored the achievement in Mexico:

1. **Strengthen governance and ownership.** Including trachoma as a priority in national and subnational public health policies and plans showed the commitment of the country to the elimination efforts. Decentralization of planning, assignment of national and subnational budgets to implement integrated intersectoral interventions for long periods, and monitoring and evaluation to guide the decision-making were key components of the trachoma elimination process. The involvement of local leaders, including indigenous authorities, was critical to sustaining efforts in the trachoma endemic municipalities.

2. **Implementation and sustainability of integrated packages of interventions with dedicated human, logistics, and financial resources.** This was key to delivering sanitation measures, personal hygiene, antibiotic treatment, and surgical corrections in chronic cases. The Trachoma Brigades were fundamental to reaching each community in the endemic municipalities with interventions for several years. The domestic investment to sustain these brigades showed the commitment of local health authorities to achieve the elimination goals.

3. **Mobilization of domestic funding** from several national and local governmental agencies including those responsible for investing in increasing access to safe water and sanitation in the trachoma endemic municipalities. This is an example of how a neglected tropical disease guided the investment of resources to improve the health interventions and living conditions of the affected populations.

4. **Community engagement in trachoma elimination efforts** Communities were involved in all efforts to eliminate trachoma. This included local leaders, families, parents and teachers in schools, NGOs, and private organizations, among others, that were engaged in actions to strengthen community awareness and disease preventive practices.

There were some challenges in the compilation of the historical information to compile the dossier, including:

**1. Gaps of historical information:** there were periods for which no records of trachoma actions were found. This might be the result of a long-lasting elimination process in a federative country with a decentralized health system. Changes in health authorities, health system reforms, and changes in public health priorities, among others, are factors that might affect the continuity of public health interventions and so the availability of information.

2. **Lack of information on the costs of the health sector interventions to achieve elimination goals**

There were no data available on the investments made by the health sector specifically for trachoma actions. For instance, it was not possible to establish the cost of the Trachoma Brigades which was one of the main strategies to implement integrated actions in the former endemic communities.

3. **Changes in the definitions of trachoma cases throughout the years**

In 1987, WHO published the simplified trachoma grading system [30] as a standardized method for clinical identification of the disease. Before it, the diagnosis was made based on different grading scales with non-standardized clinical manifestations which is a challenge to compare the trends of trachoma indicators through decades or centuries such as in Mexico. This might impose some limitations on the data analysis, but sufficient time had passed between 1987 and 2016 when Mexico submitted the dossier requesting the validation of the elimination to be confident in the indicators that supported the achievement.

After achieving the elimination, actions for trachoma were suspended for two years in Chiapas State due to the need to dedicate the Trachoma Brigades to attend other public health problems. This is a challenge, especially in a disease whose transmission has not been interrupted and reemergence is a potential risk. Mexico has not carried out post-validation surveillance mainly due to the lack of international standardized recommendations in this regard. Post-validation monitoring and surveillance are critical to detect the resurgence of the disease because the elimination as a public health problem is a reversible status. In 2020 and 2021, preventive education programs have continued in the former endemic municipalities in Chiapas as well as the active search of cases in children and surgery of detected TT cases [31]. Sustaining post-elimination activities and integrated monitoring and evaluation within the national programs have been key to success in the countries where trachoma has been eliminated such as Oman, Morocco, and Iran [32]. Surveillance in cross-border areas with Guatemala is needed. Although Guatemala reported prevalences of TF and TT below the elimination thresholds in the Sololá region, the only known endemic area and close to the border with Chiapas, trachoma surveillance on the border of the two countries has been recommended as part of the post-validation phase [33]. Also, the country has been recommended to survey the former endemic municipalities of Chiapas including trachoma examination of children and adults, collection of eye swab samples to identify the presence of *C*. *trachomatis*, and include serology to detect antibodies against the bacterium, especially in young children aged 1 to 4 years born after elimination [34]. Such a survey can contribute evidence about the current epidemiological status of those populations regarding trachoma.

Surveillance actions must be accompanied by maintaining preventive interventions, especially facial cleanliness and environmental improvements to prevent the resurgence of the disease [35]. Integrations of these actions in other public health interventions would be ideal to make them sustainable.

## Conclusions

The control and elimination of trachoma as a public health problem in Mexico is a true success story that the world can learn from. It highlights how effective coordination across state and federal health agencies, domestic investment, consistent communication, local advocacy, and strong leadership can make a difference in effective program implementation and successful rollout. However, to maintain elimination status, and to prevent the resurgence of trachoma, continued surveillance, facial cleanliness and personal hygiene actions, monitoring, evaluation, and treatment of TT cases are critical.

Lessons learned in Mexico might be useful in other trachoma endemic countries in the Americas such as Guatemala [36,37], Brazil [38], Colombia, and Peru, as well as in other regions. High-level political commitment and intersectoral collaborations to tackle key social and environmental determinants of diseases is a major lesson learned not only for the elimination of trachoma but for other neglected tropical diseases (NTD) still highly prevalent in the region [39]. This is part of the shifts proposed in the NTD road map 2021–2030 to accelerate the control and elimination of this group of diseases [40,41].
